# The Story of the Insane from Year to Year

**Published:** 1902-08-23

**Authors:** 


### THE STORY OF THE INSANE FROM YEAR TO YEAR.

FIFTEENTH ANNUAL SERIES.

### SURREY COUNTY ASYLUM AT BROOKWOOD.

ON January 1st this asylum contained 1,050 patients, and on the last day of December the numbers had fallen to 1,002; but this decrease was almost entirely owing to the transfer of cases to other asylums and not to diminution in the number of admissions, although these were 14 lower than in the year previous. There were 229 first admissions, 42 re-admissions, 96 discharged recovered, 32 relieved, 76 not improved, and 115 deaths. The average number resident during the year was 1,053, and the total number under care was 1,321. The recoveries calculated on the admissions stand at the very fair percentage of 37·69, and the deaths calculated on the average number resident at 10·92 per cent., which is rather high. Of the year's deaths 55 were caused by cerebral and spinal diseases, 15 by consumption, 9 by other lung diseases and one by enteritis. In no fewer than 94 instances the cause for insanity was attributed to such moral causes as adverse circumstances, religious excitement, and mental anxiety. Among the physical causes intemperance in drink heads the list with 40 cases, and hereditary influence follows close on it with 39. Dr. Barton says that the alcoholic cases show no tendency to diminution in his asylum, and, although the large majority make rapid recoveries, their invariable tendency is to relapse, and to eventually swell the chronic class." In other words, our paternal Government allows a man to make a beast of himself time after time until he has ruined the happiness of his family and he has become a charge on the rates as long as he lives. Dr. Barton had no case of restraint during the year, but the apparent number of seclusions has gone up considerably, because it seems that the Lunacy Commissions now require that any patient placed in a single-bedded room by day has to be recorded in the Register of seclusions, whereas formerly such return was only made when the door was locked. It would be difficult for the plain men of the earth to see what the Commissioners gain by the change, or, for the matter of that, what Dr. Barton loses by it; but it is easy enough to see that in some asylums the welfare of the patients may suffer greatly. Four attendants and five nurses have passed the examination for the nursing certificate of the Medico-Psychological Association, for which classes are regularly held during the winter months. So far so good; but it does not appear that Dr. Barton has yet instituted a three-years' course of instruction, which omission we look upon as a great drawback. We congratulate attendant King on having obtained his pension after 22 years' service as night attendant. Assuredly he deserves it. The committee say they "have pleasure in expressing their approval of the management of the asylum by the medical superintendent, Dr. Barton." The weekly charge is 12s. per head.

### EASTERN COUNTIES' ASYLUM FOR IDIOTS AT COLCHESTER.

THERE were 255 patients in residence on January 1st and 252 on December 31st. There were 23 admissions, 12 discharges, and 14 deaths. The average daily number resident was 246. To show the unfavourable nature of the material which the authorities have to work on we may mention that of the 23 admissions, 9 could not speak and 3 could not walk. Mr. Turner says, "I should naturally prefer that the subscribers should select those who are capable of receiving permanent benefit, but until public provision is made for the helpless and hopeless classes I do not feel I can urge this very strongly." It is pleasant to read such a sensible remark so temperately expressed, and while we sympathise with Mr. Turner in the hopeless nature of so much of his work we cannot help thinking of the cottage homes so often made miserable by the presence of these utterly hopeless cases. That much is done by Mr. Turner and his fellow-workers at Colchester is proved by the following figures. Of the total number of patients 129 are receiving Bible lessons, 138 attend singing classes, 184 are able to join in the entertainments, and many are taught reading, writing and arithmetic. Dr. Luard's report is an unusually able and instructive one. It deals more particularly with the great tubercle question. It is well-known that consumption is more prevalent in our county asylums than in the general population, and it is more prevalent in idiot asylums than it is in county asylums. This is the evil that Dr. Luard so clearly points out. He sees and admits the unusual difficulties which exist in carrying out preventive and curative measures in an idiot asylum; and Fabian-like he is content to rest for a while rather than adopt half measures. In the meantime warm clothing by day and by night, and plenty of ventilation will do something towards reaching the goal he seeks. We hope the asylum is not warmed by any poisonous system of hot-air or hot-water contrivances.

### EAST SUSSEX ASYLUM AT HAYWARD'S HEATH.

THERE were 967 patients in this asylum on January 1st, and at the end of the year the number had risen to 975. The total number of admissions was 224, and 43 of these were readmissions. There were 74 discharged recovered, 24 discharged relieved, 28 discharged not improved, and 90 died. The average daily number resident was 956, and the total number under treatment was 1,188. Put into percentages it is seen that the recoveries reached 36 2 calculated on the admissions, and that deaths were 9·4 on the average number resident. Of the year's deaths, 42 were caused by cerebral and spinal diseases, 2 by consumption, 15 by other lung diseases, and 1 by colitis. Table X. shows that such moral causes as domestic trouble, mental anxiety, and religion account for insanity in 36 of the cases, and among the physical causes, intemperance in drink accounts for 26, previous attacks for 53, and hereditary influence for 50. Pressure on the space was so great that a contract had to be entered into with the Nottingham City Asylum for the reception of 30 male patients. As these have to be paid for at the rate of 14s. a week each, and as the actual cost of patients in the Sussex Asylum is only 9s. 8d. a week, it follows that the Sussex ratepayers have to find £338 a year more than they ought to do from this one contract; and as other contracts are in force with the Exeter, Burntwood and West Sussex Asylums, some idea may be formed of the total loss to East Sussex. The death is mentioned of Mr. S. A. Mortlock, the well-known and efficient clerk of the asylum, who had been an officer of the institution since its opening in 1859. The committee have wisely decided to give an additional £2 a year to all attendants and nurses who hold the nursing certificate. This is becoming a common practice, and we should like to see it universal. In the discontinuance of beer as an article of diet and its substitution by an extra allowance of food, another sensible decision has been arrived at. "The committee desire to record their appreciation of the able manner in which Dr. Walker has discharged his onerous duties as medical superintendent." As already said the weekly cost per head is 9s. 8½d.

### DERBY BOROUGH ASYLUM.

On January 1st this Asylum contained 306 patients, and December 31st the number had risen to 319. There were 75 first admissions, 16 readmissions, 40 discharged recovered, 11 discharged relieved, and 21 died. The average number resident was 320, and the total number under treatment was 397. The recoveries stand at 45·1 per cent. of the admissions, and the deaths at 6 5 per cent. of the average number resident. Thirteen of the deaths were caused by cerebral and spinal diseases, 1 by consumption, and 1 by pleurisy. Of the year's admissions 26 owed their insanity to such moral causes as domestic trouble, adverse circumstances, and religion. Hereditary influence comes first among the physical causes and accounts for 34, previous attacks for 19, and intemperance in drink for 17. Dr. Macphail says that public opinion has not yet reached the stage when heredity can be considered a preventible cause of insanity. That is true, but none the less it is a preventible cause, and we look to Dr. Macphail and his *confrères* to do their best to educate public opinion to the point when the fact can be realised and acted upon. He says that intemperance "may be placed in the foreground as the chief preventible cause of insanity," and he adds that "two facts must be remembered in any deductions we are tempted to make from statistics: the one is that insanity in some cases so lessens the self-control that intemperance is the result and not the cause of the mental disease; the other is that a large proportion of our relapsed cases are alcoholic, and the same patients recur over and over again and swell the number in which alcoholic excess is tabulated as the cause." When speaking of the discharges, Dr. Macphail says that "no recovery was obtained in patients stated to have been ill for over six months before being sent to me for treatment." Dr. Macphail is discreetly silent about the recommendation of the Commissioners that a room should be provided "for the prosecution of the finer pathological research." Perhaps he thinks that it is no part of his duty to engage in that work. The Committee of Visitors say they "have pleasure in renewing the expression of their entire satisfaction with the manner in which Dr. Macphail continues to discharge the responsible and onerous duties which devolve upon him." The weekly cost of maintenance is 10s. 10d. per head.

